# Incidence and Clinical Characteristics of Herpes Zoster in Patients With Spondyloarthritis Receiving Biologic Therapy: A 36-Month Multicenter Registry-Based Study

**DOI:** 10.7759/cureus.110965

**Published:** 2026-06-16

**Authors:** Ilham Ben Marzouk, Samira Rostom, Imane El Binoune, Ghizlane Ghoullam, Bouchra Amine, Redoaun Abouqal, Ahmed Bezza, Fadoua Allali, Imane El Bouchti, Imad Ghozlani, Hasna Hassikou, Taoufik Harzy, Linda Ichchou, Radouane Niamane, Ouafa Mkinsi, Ihsane Hmamouchi, Rachid Bahiri

**Affiliations:** 1 Rheumatology A, El Ayachi Hospital, Ibn Sina University Hospital, Rabat-Sale, MAR; 2 Laboratory of Biostatistics, Clinical Research and Epidemiology, Mohammed V University, Rabat, MAR; 3 Rheumatology, Military Hospital Mohammed V, Ibn Sina University Hospital, Rabat, MAR; 4 Rheumatology B, El Ayachi Hospital, Ibn Sina University Hospital, Rabat, MAR; 5 Rheumatology, Arrazi University Hospital, Marrakech, MAR; 6 Rheumatology, Oued Eddahab Military Hospital, Faculty of Medicine and Pharmacy, Ibn Zohr University, Agadir, MAR; 7 Rheumatology, Military Hospital Moulay Ismail, Hassan II University Hospital, Meknes, MAR; 8 Rheumatology, Hassan II University Hospital, Faculty of Medicine, Pharmacy and Dental Medicine, Sidi Mohamed Ben Abdellah University, Fez, MAR; 9 Rheumatology, Mohammed VI University Hospital, Mohammed I University, Oujda, MAR; 10 Rheumatology, Military Hospital Avicenne, Mohammed VI University Hospital, Marrakech, MAR; 11 Rheumatology, Ibn Rochd University Hospital, Casablanca, MAR; 12 Faculty of Medicine, Health Sciences Research Center (CReSS), International University of Rabat (UIR), Rabat, MAR

**Keywords:** anti-tnf alpha, biological dmards, herpes zoster virus, risk factors, spondyloarthritis

## Abstract

Objectives: Herpes zoster, caused by the reactivation of varicella-zoster virus, has been reported in patients with autoimmune inflammatory diseases receiving biologic therapies. However, data regarding its occurrence in patients with spondyloarthritis (SpA) remain limited. This study aimed to determine the incidence of herpes zoster and describe the clinical characteristics of affected patients with SpA receiving biologic therapy.

Methods: A multicenter retrospective registry-based study was conducted over a 36-month period, including patients with SpA receiving biologic therapy and registered in the Moroccan Society of Rheumatology Biotherapy Registry (BRMSR). Clinical, laboratory data, including erythrocyte sedimentation rate and C-reactive protein, and therapeutic data were collected at baseline, at 36 months, and at the time of herpes zoster infection. A descriptive statistical analysis was performed.

Results: A total of 194 patients with SpA were included, with a mean age of 40.23 ± 13.68 years. The cohort included 123 males (63.4%) and 71 females (36.6%), with a mean disease duration of 11 ± 7 years. Most patients (98.5%) were treated with anti-tumor necrosis factor agents, with etanercept being the most commonly prescribed biologic therapy. The incidence of herpes zoster was 6.87 cases per 1,000 person-years (95% CI: 2.018-16.85). Four cases of herpes zoster were identified. These patients were all older than 55 years, had associated comorbidities, and had prolonged exposure to biologic therapy.

Conclusions: The incidence of herpes zoster in patients with SpA receiving biologic therapy was low. The four reported cases shared common clinical characteristics, including older age, the presence of comorbidities, and prolonged exposure to biologic therapy. Further studies with larger populations and longer follow-up are needed to better characterize herpes zoster occurrence in this population.

## Introduction

Herpes zoster is caused by the reactivation of varicella-zoster virus (VZV), a human herpesvirus responsible for primary varicella infection, which may later reactivate as herpes zoster. This reactivation occurs mainly in the setting of impaired cell-mediated immunity, particularly in older adults and immunocompromised individuals, including those receiving immunosuppressive therapies, and may be complicated by neurological manifestations [1]. The estimated lifetime risk of herpes zoster ranges from 25% to 30% and increases substantially with age, reaching nearly 50% in individuals older than 80 years [2]. Clinically, the disease typically progresses through distinct pain phases, including acute and subacute pain, followed in some cases by postherpetic neuralgia, which represents the most disabling complication and significantly impacts patients’ quality of life [2].

Spondyloarthritis (SpA) is a chronic immune-mediated inflammatory disease of unknown etiology that primarily affects young adults. It is characterized by predominant axial involvement and may be associated with various extra-articular manifestations. The management of SpA frequently relies on immunomodulatory or immunosuppressive treatments, particularly biologic disease-modifying antirheumatic drugs (bDMARDs), which have considerably improved disease outcomes [3].

While an increased risk of herpes zoster infection has been extensively reported in patients with rheumatoid arthritis (RA) [4], available data concerning patients with SpA remain limited. In particular, evidence regarding herpes zoster occurrence in individuals treated with anti-tumor necrosis factor-α agents, which are widely used in SpA, is scarce. Nevertheless, several studies suggest that patients with chronic inflammatory rheumatic diseases may present a higher susceptibility to herpes zoster when treated with conventional disease-modifying antirheumatic drugs (DMARDs) or bDMARDs, likely related to immune dysregulation and treatment-induced immunosuppression [4].

To date, no study has specifically evaluated the incidence and clinical characteristics of herpes zoster among Moroccan patients with SpA receiving biologic therapy. Therefore, the aim of the present study was to assess the incidence of herpes zoster in this population and describe the clinical characteristics of patients who developed the infection.

## Materials and methods

A multicentric retrospective population-based study was conducted. Patient data were retrieved from the Biotherapy Registry of the Moroccan Society of Rheumatology (BRMSR) [5]. The BRMSR is a multicenter historical-prospective registry that tracks patients treated for SpA and RA with biologic therapies in the 10 university rheumatology departments in Morocco.

The registry inclusion period began in May 2017 and ended in December 2018, corresponding to the first database lock, with patients followed for a duration of three years. Demographic, clinical, therapeutic, and outcome data were extracted from the registry database. To qualify for inclusion in the registry, patients had to be at least 18 years old and undergoing treatment for RA or SpA with biologic therapy, whether newly initiated or already ongoing.

Patients receiving biologic therapy for indications other than RA or SpA, as well as those with juvenile idiopathic arthritis, were excluded from the registry. A total of 419 patients were included in the registry, of whom 225 had RA and 194 had SpA.

The primary objective of the BRMSR registry was to assess the safety of biologics used in the management of RA or SpA. The secondary objectives were to evaluate the effectiveness of biologics in real-world settings and to assess the impact of biologics on patients’ quality of life. The protocol for the original BRMSR study was reviewed and approved by local institutional review boards and the national ethics committee, namely the Ethics Committee for Biomedical Research of Mohammed V University in Rabat, Faculty of Medicine and Pharmacy of Rabat.

The present study, conducted over a 36-month period, included the 194 patients with SpA identified from the BRMSR registry (patients had to be at least 18 years old and receiving biologic therapy, whether newly initiated or already ongoing) who met the Assessment of SpondyloArthritis International Society (ASAS) 2009 classification criteria [6]. Clinical, biological, and therapeutic data were collected at baseline, at 36 months, and at the time of herpes zoster infection from the BRMSR registry. Herpes zoster cases were defined as physician-confirmed dermatomal vesicular eruptions compatible with VZV reactivation and requiring antiviral treatment. The incidence of herpes zoster infection was calculated for the entire study population. All personal data were anonymized. Written informed consent had been obtained from all participants at the time of their inclusion in the BRMSR registry, according to the registry protocol approved by the Ethics Committee for Biomedical Research of Mohammed V University in Rabat, Faculty of Medicine and Pharmacy of Rabat. The present study was conducted as a retrospective analysis of anonymized registry data.

The study examined a range of clinical and diagnostic characteristics, including age, sex, disease duration, family history of SpA, peripheral arthritis, inflammatory back pain, and enthesis tenderness. Radiological evaluations such as X-rays, magnetic resonance imaging, and power Doppler ultrasonography were analyzed in conjunction with biological markers, including erythrocyte sedimentation rate (ESR), C-reactive protein (CRP), and HLA-B27 status.

Disease activity was assessed using validated measures, including the Bath Ankylosing Spondylitis Disease Activity Index (BASDAI) [7], the Ankylosing Spondylitis Disease Activity Score with C-reactive protein (ASDAS-CRP) [8], and the Bath Ankylosing Spondylitis Functional Index (BASFI) [9].

Additionally, comorbidities such as depression, diabetes, fibromyalgia, latent tuberculosis, dyslipidemia, and osteoporosis were documented. Extra-articular manifestations, including uveitis, psoriasis, and inflammatory bowel disease, were also evaluated. Finally, data on therapeutic interventions, including the use of non-steroidal anti-inflammatory drugs, analgesics, corticosteroids, and conventional synthetic DMARDs, were analyzed to provide a comprehensive overview of treatment patterns.

The statistical approach was primarily descriptive, aiming to characterize the overall cohort and provide a detailed description of patients who developed herpes zoster infection during follow-up. The statistical analysis was limited to descriptive methods, consistent with the study objective of estimating the incidence of herpes zoster and describing the clinical characteristics of affected patients within this population.

The study complied with ethical guidelines, including the Declaration of Helsinki and good clinical practice standards.

## Results

Study population

A total of 194 patients with SpA were included in the study (Table 1). The mean age was 40.23 ± 13.68 years, and there were 123 males (63.4%) and 71 females (36.6%), with a male-to-female ratio of 1.73. The mean disease duration was 11.21 ± 7.01 years.

**Table 1:** Sociodemographic, clinical, and paraclinical characteristics of the study population a, mean and SD; b, number and percentage TNF: tumor necrosis factor.

Parameter	Total (n=194)
Ageᵃ (years)	40.23 ± 13.68
Gender ratio (M/F)	1.73
Hypertensionᵇ	11 (5.7)
Diabetesᵇ	10 (5.2)
Heart diseaseᵇ	2 (1.0)
Smokingᵇ	21 (10.8)
Disease durationᵃ (years)	11.21 ± 7.01
Axial involvementᵇ	186 (95.9)
Peripheral involvementᵇ	134 (69.1)
Enthesitic involvementᵇ	115 (59.3)
Radiographic formᵇ	170 (87.6)
Psoriasisᵇ	13 (6.7)
Chronic inflammatory bowel diseaseᵇ	20 (10.3)
Uveitisᵇ	27 (13.9)
Corticosteroid therapyᵇ	43 (22.2)
Duration of exposure to biologicsᵃ (years)	5.68 ± 2.39
Anti-TNF therapyᵇ	191 (98.5)
Infliximabᵇ	49 (25.3)
Adalimumabᵇ	59 (30.4)
Golimumabᵇ	19 (9.8)
Etanerceptᵇ	64 (33.3)
Certolizumabᵇ	3 (1.5)

Regarding disease characteristics, axial involvement was present in 186 (95.9%) patients, peripheral involvement in 134 (69.1%) patients, and enthesitic involvement in 115 (59.3%) patients. A radiographic form of the disease was observed in 170 (87.6%) patients. Comorbidities included hypertension in 11 (5.7%) patients, diabetes in 10 (5.2%) patients, heart disease in 2 (1%) patients, and smoking in 21 (10.8%) patients. Extra-articular manifestations included psoriasis in 13 (6.7%) patients, chronic inflammatory bowel disease in 20 (10.3%) patients, and uveitis in 27 (13.9%) patients.

Regarding treatment, 43 (22.2%) patients were receiving corticosteroids. Almost all patients (191; 98.45%) were treated with anti-tumor necrosis factor agents. The most frequently prescribed biologic was etanercept (64; 33.3%), followed by adalimumab (59; 30.4%), infliximab (49; 25.3%), golimumab (19; 9.8%), and certolizumab (3; 1.5%). No patients received anti-interleukin-17 or Janus kinase inhibitors during the study period.

Characteristics of patients with herpes zoster

Among the 194 patients receiving biologic therapies, four cases of herpes zoster infection were identified, corresponding to an incidence rate of 6.87 cases per 1,000 patient-years (95% CI: 2.18-16.85). The detailed sociodemographic and clinical characteristics of these patients are presented in Table 2.

**Table 2:** Sociodemographic, clinical, and paraclinical characteristics of patients with herpes zoster SpA: spondyloarthritis.

Variable	Case 1	Case 2	Case 3	Case 4
Age (years)	61	64	55	65
Sex	Male	Male	Female	Female
Disease duration (years)	13	10.5	5	23.8
Axial involvement	Yes	Yes	Yes	No
Peripheral involvement	Yes	Yes	Yes	Yes
Enthesitis	No	Yes	Yes	No
Radiographic SpA	Yes	Yes	Yes	No
HLA-B27 positive	Yes	Yes	No	No

The ages of patients with herpes zoster ranged from 55 to 65 years. Two patients were male and two were female. Disease duration ranged from 5 to 23.8 years. Axial involvement was observed in three of the four patients, all of whom also had peripheral involvement. Enthesitis was present in two cases, three patients had a radiographic form of SpA, and two were HLA-B27 positive.

Clinical evolution of herpes zoster cases

The evolution of clinical and paraclinical parameters between baseline and the 36-month follow-up is shown in Table 3. At baseline, one patient had arterial hypertension. During follow-up, two additional patients developed comorbidities, including type 2 diabetes and dyslipidemia.

**Table 3:** Comparison of clinical and paraclinical characteristics of patients with herpes zoster at baseline and 36 months ASDAS-CRP: Ankylosing Spondylitis Disease Activity Score-C-reactive protein; BASDAI: Bath Ankylosing Spondylitis Disease Activity Index; BASFI: Bath Ankylosing Spondylitis Functional Index; CRP: C-reactive protein; csDMARD: conventional synthetic disease-modifying antirheumatic drug; ESR: erythrocyte sedimentation rate.

Variable	Case 1, M0	Case 1, M36	Case 2, M0	Case 2, M36	Case 3, M0	Case 3, M36	Case 4, M0	Case 4, M36
Comorbidities	None	None	Smoking (26 pack-years)	None	Hypertension	Hypertension	Dyslipidemia	None
Corticosteroid therapy	Past corticosteroid therapy (stopped in 2010)	None	None	None	None	None	None	None
csDMARDs	Methotrexate, stopped	None	Methotrexate, stopped	None	Methotrexate, stopped	None	Sulfasalazine	None
Line of biologic therapy	Second-line	Second-line	Second-line	Second-line	First-line	First-line	Third-line	Third-line
Biologic therapy	Etanercept	Etanercept	Etanercept	Etanercept	Etanercept	Etanercept	Etanercept	Etanercept
ESR (mm/h; normal <20)	102	—	5	61	15	33	17	10
CRP (mg/L; normal <5)	62	8	3.2	58	1.86	2.92	1.77	5
ASDAS-CRP	4.5	2.1	1.6	3.6	1.1	—	1.3	0.9
BASDAI	4.8	0.9	1.8	4.2	0.7	—	1.0	4.0
BASFI	1.4	1.2	1.3	2.1	1.2	—	1.5	5.0

Biological inflammatory markers showed variability between baseline and follow-up. ESR values ranged from 5 to 102 mm/h, and CRP values from 1.77 to 62 mg/L. Disease activity scores also varied: BASDAI ranged from 0.7 to 4.8, ASDAS-CRP from 0.9 to 4.5, and BASFI from 1.2 to 5 during follow-up.

Profile at the time of herpes zoster infection

At the time of herpes zoster infection (Table 4), all four patients were receiving etanercept. Previous biologic exposure included adalimumab and infliximab in some cases. Duration of exposure to biologic therapy ranged from 5 to 14 years.

**Table 4:** Clinical, biological, and therapeutic characteristics of patients with herpes zoster at the time of infection ASDAS-CRP: Ankylosing Spondylitis Disease Activity Score-C-reactive protein; BASDAI: Bath Ankylosing Spondylitis Disease Activity Index; BASFI: Bath Ankylosing Spondylitis Functional Index; CRP: C-reactive protein; ESR: erythrocyte sedimentation rate.

Variable	Case 1	Case 2	Case 3	Case 4
Time of infection (months after biologic initiation)	30	12	18	30
Comorbidities	None	Hypertension	Hypertension, type 2 diabetes	Dyslipidemia
Current biologic therapy	Etanercept	Etanercept	Etanercept	Etanercept
Previous biologic therapies	Adalimumab	Adalimumab	None	Infliximab, adalimumab
Duration of biologic exposure (years)	11	9	5	14
ESR (mm/h; normal <20)	10	10	15	28
CRP (mg/L; normal <5)	2.89	12.35	1.41	1.98
ASDAS-CRP	2.1	1.7	1.5	2.7
BASDAI	1.8	0.2	2.0	3.7
BASFI	1.1	0.4	—	3.9

At the time of infection, ESR ranged from 10 to 28 mm/h, CRP from 1.41 to 12.35 mg/L, BASDAI from 0.2 to 3.7, ASDAS-CRP from 1.5 to 2.7, and BASFI from 0.4 to 3.9. Three of the four patients had comorbidities during the herpes zoster episode.

## Discussion

Our study suggested a low incidence of herpes zoster infection in patients with SpA, with a rate of 6.87 cases per 1,000 person-years. A common clinical observation among the four patients who developed herpes zoster was an age greater than 55 years, the presence of comorbidities, and biologic therapy exposure exceeding five years. In comparison with global incidence rates, the incidence of herpes zoster observed in our study aligns closely with rates reported in Taiwan, ranging from 4.04 to 6.24 cases per 1,000 person-years [10,11]. By contrast, the incidence in the United States and Europe is slightly lower, ranging from 3.2 to 3.7 cases per 1,000 person-years [12,13]. Regional differences in herpes zoster incidence remain poorly understood but may be influenced by ethnic, genetic, and cultural factors [14]. In patients with SpA, the incidence of herpes zoster has been reported to be higher than in the general population in the United States, although the difference was not statistically significant [15]. Conversely, studies from South Korea have shown comparable incidence rates of herpes zoster between patients with SpA and the general population, with rates of 11.0 and 10.04 cases per 1,000 person-years, respectively [16,17].

Age has consistently been identified as a significant risk factor for herpes zoster infection, likely due to immunosenescence and the decline in varicella zoster virus-specific memory CD4+ cells among older individuals [18-20]. This is consistent with findings from Taiwan [21] and is corroborated by our study, as all herpes zoster cases occurred in patients aged over 55 years.

Cancer is another established risk factor for herpes zoster, with affected patients experiencing a two- to five-fold higher incidence than the general population, as reported by Rusthoven et al. [22] and Dunst et al. [23]. Similarly, immunosuppressive therapies, including corticosteroids and certain DMARDs, may contribute to an elevated risk of herpes zoster. However, the association between these treatments and herpes zoster remains controversial. While some studies suggest an increased risk with both conventional synthetic DMARDs and bDMARDs [24,25], others have found no such relationship [26].

Combination therapy involving anti-tumor necrosis factor agents and conventional synthetic DMARDs has been shown to significantly increase herpes zoster risk, as highlighted by Zisman et al. [27].

A high incidence of herpes zoster has also been reported in other autoimmune conditions, such as systemic lupus erythematosus and RA, compared with the general population [28,29]. These observations suggest that the increased susceptibility to herpes zoster may not be specific to SpA but may reflect a broader effect of immune dysregulation and exposure to immunosuppressive therapies, including glucocorticoids, conventional synthetic DMARDs, and bDMARDs.

Effective prevention of herpes zoster in patients with SpA relies mainly on vaccination before initiating biologic therapy, according to current recommendations. In the event of herpes zoster, particularly in immunocompromised patients, prompt initiation of antiviral therapy remains an essential component of management [30]. However, the role of herpes zoster vaccination specifically in patients with SpA receiving immunomodulatory treatments requires further investigation through large-scale multicenter studies.

Strengths and limitations

The primary strength of our study lies in its multicenter design, with data derived from a national registry (BRMSR). Additionally, this is the first study in the region to investigate herpes zoster infections in patients with SpA treated with biologic therapies.

However, several limitations must be acknowledged. The relatively small sample size, the three-year follow-up period, and the recruitment of patients exclusively from tertiary centers may limit the generalizability of our findings. Additionally, the retrospective design of this study may constitute a limitation due to the inherent constraints of retrospective analyses. Future studies should focus on larger, more representative populations and extend follow-up durations to provide more robust data.

## Conclusions

The incidence of herpes zoster among patients with SpA receiving biologic therapy was 6.87 cases per 1,000 person-years in our cohort over a 36-month follow-up period. The reported cases of herpes zoster shared common clinical characteristics, including older age and the presence of comorbidities.

Our results highlight the importance of preventive strategies in patients with SpA treated with biologic therapies. Herpes zoster vaccination should be considered in patients at increased risk, particularly older individuals and those with comorbidities. Careful clinical monitoring and prompt initiation of antiviral therapy may contribute to reducing herpes zoster-related complications.
